# The coevolution of cooperation and dispersal in social groups and its implications for the emergence of multicellularity

**DOI:** 10.1186/1471-2148-8-238

**Published:** 2008-08-19

**Authors:** Michael E Hochberg, Daniel J Rankin, Michael Taborsky

**Affiliations:** 1 Institut des Sciences de l'Evolution, Centre National de la Recherche Scientifique, UMR 5554, Université Montpellier II, 34095 Montpellier, France; 2 Santa Fe Institute, 1399 Hyde Park Road, Santa Fe, NM 87501, USA; 3 National Centre for Ecological Analysis and Synthesis, 435 State Street, Suite 300, Santa Barbara, CA 93101-3351, USA; 4 Department of Behavioural Ecology, University of Bern, Wohlenstr. 50a, 3032 Hinterkappelen, Switzerland

## Abstract

**Background:**

Recent work on the complexity of life highlights the roles played by evolutionary forces at different levels of individuality. One of the central puzzles in explaining transitions in individuality for entities ranging from complex cells, to multicellular organisms and societies, is how different autonomous units relinquish control over their functions to others in the group. In addition to the necessity of reducing conflict over effecting specialized tasks, differentiating groups must control the exploitation of the commons, or else be out-competed by more fit groups.

**Results:**

We propose that two forms of conflict – access to resources within groups and representation in germ line – may be resolved in tandem through individual and group-level selective effects. Specifically, we employ an optimization model to show the conditions under which different within-group social behaviors (cooperators producing a public good or cheaters exploiting the public good) may be selected to disperse, thereby not affecting the commons and functioning as germ line. We find that partial or complete dispersal specialization of cheaters is a general outcome. The propensity for cheaters to disperse is highest with intermediate benefit:cost ratios of cooperative acts and with high relatedness. An examination of a range of real biological systems tends to support our theory, although additional study is required to provide robust tests.

**Conclusion:**

We suggest that trait linkage between dispersal and cheating should be operative regardless of whether groups ever achieve higher levels of individuality, because individual selection will always tend to increase exploitation, and stronger group structure will tend to increase overall cooperation through kin selected benefits. Cheater specialization as dispersers offers simultaneous solutions to the evolution of cooperation in social groups and the origin of specialization of germ and soma in multicellular organisms.

## Background

Cooperation is central to transitions in individuality [1-4]. Full individuality is achieved when components cooperate and relinquish their autonomy to the larger whole. Depending on the type of transition, this may necessitate the division of labor in growth, reproduction, development, feeding, movement, and protection against external aggression and internal conflict [5,6]. In the evolution of multicellularity, the chain of events from autonomous individuals at one level to the incorporation of these individuals into a more complex entity remains unclear [5]. However, some of the putative forces are likely to be general, since multicellularity has arisen many different times in evolutionary history [7,8]. Moreover, that many groupings do not show sophisticated specialization and are characterized by substantial levels of internal conflict [9,10], suggests that incomplete multicellularity may be a frequent outcome. What mechanisms are essential to generate individuality? We believe that a general theory needs to explain both full and incomplete transitions towards multicellular individuals.

Previous work highlights group and kin selection [5,10,11], organism size [12,13], and the reorganization of fitness and specialization tradeoffs [14] as playing roles in the evolution of multicellularity. A feature common to these mechanisms is the establishment and maintenance of cooperative behaviors amongst subunits through, for example, conflict mediation (e.g. [15,16]). Based on a recent literature review, Grosberg and Strathmann [8] argued that for cooperation to emerge and favor the specialization of subunits, groups of cells need to reduce genetic conflicts arising in cell lineages [10]. They conclude that several mechanisms can limit such conflicts, perhaps the most important being development from a single cell (e.g., [5,16]).

A key type of subunit specialization in multicellular organisms is the separation of germ and soma [1,5,10,17,18]. Separating germ and somatic functions amongst individual cells or cell lineages requires that each sacrifice autonomy. Theory predicts that such specialization is promoted by non-mutually exclusive mechanisms such as cooperation and relatedness amongst cell lineages [10], cheater control [1,19,20] and adaptive responses to tradeoffs between survival and reproductive functions, i.e. a covariance effect augmenting the fitness of the group over the average fitness of its members [14]. It is not known whether the alignment of fitness interests in emerging soma and germ lines tends to occur before, during or after other types of specialization characteristic of multicellular organisms [12].

A pervasive feature in a diverse array of social systems is that individuals not contributing to the common good either act as dispersers, or are either rewarded for, or coerced into, cooperating. Examples range from bacteria (e.g. *Pseudomonas fluorescens*) through protozoa (e.g. *Volvox carteri*) to metazoans, like eusocial insects and mammals (see Additional file 1). For example, in naturally occurring *Dictyostelium *slime molds prespores secrete a chlorinated hexaphenone (DIF-1) inhibiting redifferentiation of prestalk cells into prespores, which would transpose them from "cooperative" stalk building to "cheating" spore production (i.e. a transition into the dispersing and perennial germ line; [21,22]). Cheating is further curtailed by pleiotropic effects of a gene required to permit receipt of this signal, which affects also the probability of spore formation [23]. In tunicates such as *Botryllus schlosseri*, natural chimeras consisting of genetically nonhomogenous organisms often show reproducible germ cell parasitism that is sexually inherited, with "parasitic forms" being expressed only in the germ line, i.e. in the dispersing entities [24]. In the cooperatively breeding cichlid fish *Neolamprologus pulcher*, brood care helpers of both sexes are forced to pay rent for being tolerated in a safe territory [25,26]. To avoid being punished they preemptively appease dominants by cooperative and submissive behavior [27]. Typically, in these cichlids and in cooparatively breeding meerkats *Suricata suricatta*, subordinates preparing for dispersal reduce helping [28,29], which might be explained by reduced costs of potential punishment by eviction [30,31]. In eusocial mole rats (*Heterocephalus glaber *and *Cryptomys damarensis*) non-reproductive helpers and hardly helping dispersers coexist [32-34]. Policing of subordinates by dominant breeders may simultaneously maintain social order and stimulate cooperative behaviors [35,36]. This distinction of roles between individuals is particularly obvious in the separation between soma and germ that has apparently evolved many times independently [7]. Nevertheless, there are examples where cooperative behaviors are associated with enhanced group dispersal (see Additional file 1). For example, in the soil-dwelling social bacterium *Myxococcus xanthus*, individualistic cell movement ('A-motility') promotes swarming on hard surfaces, whereas swarming on soft surfaces is a group function driven primarily by individually costly S-motility [37].

These empirical patterns merit explanation, and we take a first step by employing optimization techniques to evaluate the conditions leading to associations between dispersal and social strategy. Sociality in our models takes the form of cooperation in the production of a public good. Previous study of public goods has shown how cheating, if left unchecked, potentially leads to a "tragedy of the commons" [38,39], whereby individual selection tends to favor exploitation of the public good at some concurrent or future detriment of the group. Several non-mutually exclusive mechanisms may promote cooperation and group persistence, including kin selection (e.g., [40-42]), rewards and sanctions (e.g., [43,44]), spatial and network structure (e.g., [45-47]), and signals involving kin or non-kin (e.g., [48-50]). Recent reviews and perspectives can be found in Crespi [51], Sachs and colleagues [52], Lehmann and Keller [53], and West and coworkers [54].

We develop a model based on kin selection that incorporates dispersal specialization, as suggested by the case studies in Table S1 (see Additional file 1). We employ the terms "soma" and "germ" to represent the functions of within-group growth and dispersal leading to the founding of new groups, respectively. Our use of the terms "cooperators" and "cheaters" refers to social behaviors within the commons (e.g., soma), and this should be distinguished from the frequent usage of "cheaters" as cooperative somatic lineages trying to gain access to germ line (e.g., [1,5,8,10,22]). Specifically, cooperators contribute to the public good within a distinct group at an individual cost, and cheaters exploit the public good. Cooperators and/or cheaters may be selected to either remain in a group, or to disperse (potentially founding new groups). Our theory proposes a mechanism leading to high overall cooperation, based on dispersal specialization. In addition to increasing our understanding of cooperative and dispersal behaviors, it could apply to the evolution of multicellularity in a range of contexts, including physiologically integrated organisms [55,56], organisms with both solitary and integrated life-styles (e.g., [57]), and complex societies [58].

## Methods

We formalize our verbal arguments given above by developing and analyzing a model of coevolution between exploitation of the commons and dispersal. From the outset, we stress that our model is a highly simplified representation of this process, and not aimed to make quantitative predictions for any given system. Rather, our goal is to identify the qualitatively important drivers in the coevolution of individual strategies and the evolution of multicellularity.

In our model the focal units of selection are individuals themselves, rather than the higher-level unit. A transition to multicellularity is favored when the interests of the individual and the higher-level (the group) are aligned [5,8,15]. Previous models investigating the transition to multicellularity invoke a framework where the group is the focal unit of selection (see, for example [15]). However, focusing on the higher-level as the focal unit does not easily allow the investigation of optimization at the lower level [59], and the individually-selected conditions leading to a major transition [60]. Grosberg and Strathmann [8] have argued that many of the requirements for transitions to multicellularity exist in unicellular organisms (for social groups, see [61]). Once a transition is in progress, and the "group" begins to behave as an individual entity, one can begin to treat this unit as an evolving individual in itself.

We analyze an optimization model that takes into account the effect of both the phenotype of the focal individual and the average phenotype of the group in which it lives, on the fitness of the focal individual (see Table 1 for descriptions of parameters and variables). The approach is based on the direct fitness method [42,62] in that, by considering the effects of both individual and average group phenotypes on the fitness of a focal individual, we can apply the Price Equation to partition these effects as weighted by the relatedness of the focal individual to other members of the group [42]. We can then assess the relative impacts of (1) costs and benefits of individual behaviors and (2) kin structure, on associations between exploitative strategy within a group, and dispersal to found new groups. Nevertheless, our model oversimplifies the complexity of social behavior and dispersal decisions (for review, see [63]), and should thus be viewed as a preliminary attempt to identify patterns.

**Table 1:** Parameters and variables used in this study.

w	Individual fitness
r	Relatedness between any two randomly selected individuals in the group
s	Individual cost to cooperator growth in the group
k	Number of individuals in a group (an inverse measure of kin selection)
c	Individual cost to cooperator dispersal
e	Individual cost to cheater dispersal
Q	Impact of sedentary cheaters on the individual fitness of group members (via consumption of the public good)
P	Impact of sedentary cooperators on the individual fitness of group members (via production of the public good)
n	Relative frequency of cooperators in the group (1-n is the proportion of cheaters)
z	Relative frequency of cheaters dispersing
y	Relative frequency of cooperators dispersing
d	Overall investment in dispersal. d = yn + z(1-n)
Φ	Overall cooperation with respect to the public good. Φ = n*(1-y*)+(1-n*)z*
σ	Association between dispersal and cooperation. σ = y/(y+z)

Our model makes several assumptions. First, we do not explicitly consider dynamics, such as group founding, group numbers, individual emigration and immigration, and competition for limiting resources within or between groups. Rather, we assume negligible variation in inter-group competition. Second, our model does not explicitly incorporate genetic polymorphisms, meaning that the heritable traits are probabilities to adopt alternatives of each strategy (disperse or stay; cooperate or cheat) depending on environmental and/or social conditions [1,10,32,64-66]. Third, there is a simple direct tradeoff between an individual's viability (growth, survival and reproduction) within the group and its ability to disperse and found new groups. This is based on the well established life-history trade-off between reproduction and dispersal (see [67]), probably best studied in insects (on the physiological scale e.g. [68-70]; on the ecological scale e.g. [71,72]). Whereas growth and reproduction within the group impacts the production and consumption of the public good, the tendency to disperse reduces these impacts because of the limited presence of dispersers in the source group.

### Life cycle and fitness equations

We assume that a group's life-cycle has three sequential stages: colonization, growth, reproduction and survival of individuals within the group; exhaustion of resources; and the dispersal of survivors. Some of the survivors may stay at the same site of the source group, and others disperse as colonists to other sites.

The model tracks the fitness contribution of a mutant individual *i*, within group *j *[42,62]. Fitness effects are partitioned between cooperators and cheats–who have positive and negative impacts on the public good, respectively–and amongst dispersal strategies. Thus four strategies are possible: (1) cooperate and remain in group, (2) cooperate and disperse, (3) cheat and remain in group, and (4) cheat and disperse. Only the first and third strategies affect the public good.

The proportion of cooperators in the group is *n*_*i *_(for simplicity, hereafter we denote individual *i *within group *j *using the subscript *i *only), which can take continuous values between 0 and 1. Moreover, our model incorporates two dispersal strategies based on whether the dispersing individual is a cooperator or a cheater. We define *y*_*i *_as the investment of a cooperator in dispersal and *z*_*i *_as the investment of a given cheater in dispersal. Both of these quantities take on continuous values between zero and one. The mean proportions of dispersing cooperators and cheaters in group *j* are *y*_*j*_*n*_*j *_and *z*_*j*_(1-*n*_*j*_), respectively and overall investment in dispersal is *d*_*j *_= *y*_*j*_*n*_*j *_+ *z*_*j*_(1-*n*_*j*_).

The fitness equation takes the form

(1) *w*_*i *_= *D*(*n*_*i*_, *y*_*i*_, *z*_*i*_) *E*(*n*_*i*_, *y*_*i*_, *z*_*i*_) *G*(*n*_*i*_, *y*_*i*_, *z*_*i*_),

where the functions *D *and *E*, respectively, represent the contribution of selection on dispersal and the exploitation of the public good of individual *i *in group *j *to its own fitness. Function *G *is the overall investment in the public good in group *j*.

Dispersal is modeled by considering the fitness contributions of both individuals that stay at the site previously occupied by the group and others that disperse [73]. We assume that the costs of dispersal may differ between cooperators (*c*) and cheaters (*e*). Small costs would indicate abundant new sites for group establishment and high disperser survival. Although we consider different cases in the analysis, our general expectation is that the costs of cooperation will extend to dispersal, such that *c *> *e*.

The function, *D*, takes the form

(2) *D*(*n*_*i*_, *y*_*i*_, *z*_*i*_) = [(1 - *z*_*i *_(1-*n*_*i*_) - *y*_*i *_*n*_*i*_)/(1 - *z*_*j *_(1-*n*_*j*_) - *y*_*j *_*n*_*j *_+ (1-*e*) *z *(1-*n*) + (1-*c*)*y n*)] + [((1-*e*) *z*_*i *_(1-*n*_*i*_) + (1-*c*)*y*_*i *_*n*_*i*_)/(1 - *e z *(1-*n*) - *c y n*)].

The first term in square brackets describes the fitness of a non-disperser (1 - *z*_*i *_(1-*n*_*i*_) - *y*_*i *_*n*_*i*_) relative to the average non-disperser (1 - *z*_*j *_(1-*n*_*j*_) - *y*_*j *_*n*_*j*_) and immigrants ((1-*e*) *z *(1-*n*) + (1-*c*)*y n*). The second term describes the fitness of a disperser ((1-*e*) *z*_*i *_(1-*n*_*i*_) + (1-*c*)*y*_*i *_*n*_*i*_) given the competition it faces with residents (1 - *z *(1-*n*) - *y n*) and migrants ((1-*e*) *z *(1-*n*) + (1-*c*)*y n*) in another group. The terms *n*, *z *and *y *(i.e., without subscripts) are population-wide means. The denominator in both terms represents the amount of competition faced either in the original group, in the case of a non-disperser, or in a new group, in the case of the disperser. Note that in the limit of no dispersal, individual fitness can still be positive under the assumption that groups survive indefinitely.

All non-dispersing individuals are selected to exploit, but given our assumption that there is a cost of cooperation (*s*), this will weight selection to favoring cheaters, all else being equal. The function, *E*, describes the contribution of individual *i *to its own fitness through exploitation of the public good and is given by

(3) *E*(*n*_*i*_, *y*_*i*_, *z*_*i*_) = [(1-*z*_*i*_) (1-*n*_*i*_) + (1-s) (1-*y*_*i*_) *n*_*i*_]/[(1-*z*_*j*_) (1-*n*_*j*_) + (1-s) (1-*y*_*j*_) *n*_*j*_],

where the subscript *j *indicates mean group levels, and the constant *s *measures the cost to individual cooperators in producing the public good.

The overall effect of group investment in the public good on individual fitness is described by

(4) *G*(*n*_*i*_, *y*_*i*_, *z*_*i*_) = 1 + P (1-*y*_*j*_) *n*_*j *_- Q (1-*z*_*j*_) (1-*n*_*j*_),

where it is assumed that non-dispersing cooperators have a positive effect on the public good (scaled by P) as their frequency, *n*_*j*_, increases [74,75], whereas cheaters have a net negative effect on the public good (scaled by Q) as their frequency, 1-*n*_*j*_, increases. Note that in the absence of cooperators, cheats can persist as long as their impact on the commons is sufficiently low (*z *Q< 1). Alternatively, when group effects are nil (i.e. P = Q = 0), the notion of a group is a collection of autonomous individuals.

### Relatedness and numerical simulation methods

We analyze the model by employing the Price Equation, which enables us to express possible fitness maxima as a function of constant parameters and variables, and the relatedness, *r*, between individuals. Taylor and Frank [62] give methods for finding the equilibrium, such that for any trait *v *we have

(5) d*w*_*i*_/d*v*_*i *_= ∂*w*_*i*_/∂*v*_*i *_+ *r *∂*w*_*i*_/∂*v*_*j*_

from which we can find a steady state(s) when d*w*_*i*_/d*v*_*i *_= 0 to find any or all v* = y*, z*, n*.

In our model, *r *can either be a parameter (referred to as an "open model" by Gardner and West [76]) or can emerge from the underlying structure of the population (referred to as a "closed" model in [76]). In the latter case, we may derive *r *from the dispersal of individuals in the population with the recursion relation (e.g., [39,76])

(6) *r*(t+1) = 1/*k *+ (*k *- 1)/*k *(1 - *d*)^2 ^*r*(t).

This recursion tracks the probability that a given focal individual is identical by descent to another randomly picked individual at time *t*. The parameter *k *is the effective number of individuals in the group, and can be viewed as a measure of genetic diversity due to individual aggregation in group founding and habitat structure. [Note however that our model does not explicitly track the actual number of individuals in the group]. Low *k *is indicative of group founding by single individuals, group resistance to immigration, and abundant open sites for group founding [10,77].

In the recursion above, the term 1/*k *represents the probability that the randomly picked individual is the focal individual itself. The second term represents the probability that the randomly picked individual is different to the focal individual, and that neither have dispersed (represented by (1-*d*)^2^). This is multiplied by the relatedness from the previous round. Solving this recursion relation yields the equilibrium relatedness, which is

(7) *r *= 1/(*k *- (*k *- 1) (1 - *d*)^2^).

As we assume weak selection, the probability that a given individual disperses depends on the probability that it is a cooperator and disperses, plus the probability that it is a cheater and disperses, so *d *= *yn*+*z*(1-*n*) in this case. Under the assumptions of weak selection, we evaluate this recursion for the case when *v*_*i *_= *v*_*j *_= *v*, where *v *is the trait in question.

Optimal strategies were solved numerically. This consisted of iterating equation (5) with steps of 0.05 or smaller for a total of 100,000 steps, which was sufficient to identify the steady state in all cases. We found that whereas initial levels of evolving variables did not affect the optimal solution when only dispersal frequencies *y *and *z *evolved, initial conditions could indeed affect the optimal solution when all three variables evolved. Closer examination showed that alternative stable states were possible, one with either all cheaters (n* = 0) or all cooperators (n* = 1), and a second with both strategies persisting (0 <n* < 1). Although we cannot exclude the existence of alternative interior equilibria, our numerical studies always yielded at most a single interior solution.

## Results

We consider two scenarios. In the first (Model 1) only dispersal in cooperators (*y*) and cheaters (*z*) evolves, but not cooperation (*n*). This situation would be obtained if mechanisms not explicitly included in the model (e.g., policing, [44]) controlled the level of cooperation, or if the frequencies of cooperative behaviors were either not subject to evolution, or labile to it over much longer time scales than dispersal. More generally however, empirical study suggests that cooperative behaviors are subject to selection [78-80] and we consider the case (Model 2) in which dispersal and the frequency of cooperators (*n*) and cheaters (1-*n*) co-evolve.

In addition to optimal levels of dispersal (Model 1), and of cooperation and dispersal (Model 2), we examine the effects of model parameters on dispersal specialization σ = y*/(y*+z*), and for Model 2 only, overall cooperation Φ = n*(1-y*)+(1-n*)z* (i.e., the sum of cooperators not dispersing and of cheaters dispersing). Note that when σ = 0 (or σ = 1), although all cooperators (cheats for σ = 1) are sedentary it is not necessarily true that all cheats (cooperators for σ = 1) disperse.

### Model 1

**Figure 1:**
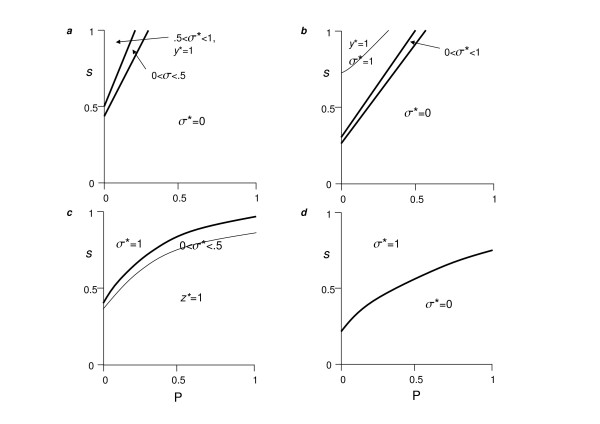
**Globally optimal associations in dispersal and exploitation strategy for Model 1**. Axes: *P *measures the impact of the public good on individual fitness, and *s *is the individual cost to cooperators in contributing to the public good. σ * = y*/(z* + y*) indexes the tendency of cooperators to disperse (σ* > 0.5) or cheats to disperse (σ* < 0.5). Thick curves demarcate areas of parameter space yielding different levels of σ, whereas thin lines show areas in with either y* = 1 or z* = 1. Caption *a*: *k *= 1.2, *n *= 0.1; caption *b*: *k *= 10, *n *= 0.1; caption *c*: *k *= 1.2, *n *= 0.9; caption *d*: *k *= 10, *n *= 0.9. Note that for legibility, very thin areas parallel to thick lines are omitted, in which 0.5 < σ* < 1 for caption *c*, and 0 < σ* < 1 for caption *d*. Unless otherwise noted, dispersal rates are greater than zero and less than unity. Other parameters: *c *= *e *= 0.2, *Q *= 0.2. See main text for numerical methods.

Optimal solutions always yielded partial or complete specialization, with cooperators tending to disperse more than cheaters (i.e., σ > 0.5) for high costs of cooperation (*s*) compared to public good's effect (*P*), and low cooperator frequencies (*n*) (Figure 1). The reverse trends promote relative cheater dispersal (σ < 0.5; Fig. 1). The impact of effective group size (*k*) is more complex. Higher *k *tends to polarize dispersal to either cooperators (y* > 0, z* = 0) or cheaters (y* = 0, z* > 0), and increases the parameter space in which cooperators dominate dispersal (areas with σ * = 1; Fig. 1).

**Figure 2:**
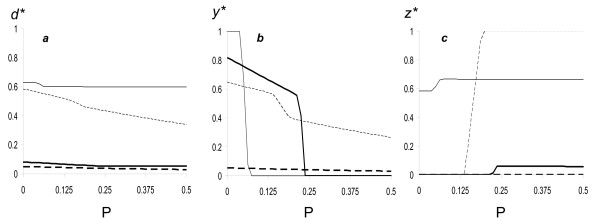
**Effects of parameters on optimal dispersal levels for Model 1**. Effects of public good production (*P*), frequency of cooperators (*n*) and effective group size (*k*). Caption *a*: overall dispersal d*; Caption *b*: investment in cooperator dispersal y*; Caption *c *investment in cheater dispersal z*. Thin line: *k *= 1.2, *n *= 0.1; dashed line: *k *= 10, *n *= 0.1; thick line: *k *= 1.2, *n *= 0.9; thick dashed line: *k *= 10, *n *= 0.9. Other parameters: *c *= *e *= 0.2, *Q *= 0.2, *s *= 0.6.

Low effective group size (low *k*) should positively associate with kin competition, and in agreement with previous work [81,82], we find that low *k *is associated with higher overall dispersal, d* (Figure 2a). Not surprisingly, d* increases with lower cooperator frequencies (*n*) and public good effects (*P*) (Fig. 2a). However, the effects of *k *and *n *on the separate cooperator (y*) and cheater (z*) dispersal frequencies are more complex (Figs. 2b, c). In particular, low *k *was always found to drive cheaters to disperse (Fig. 2c), whereas the effect on cooperators depended strongly on cooperator frequency (*n*) and public good productivity (*P*) (Fig. 2b).

Cheater and cooperator dispersal can be understood as follows. When the group is dominated by cheaters (low *n*) and production of the public good (*P*) is small, increasing cooperator sedentariness (1-y*) has little beneficial effect on fitness (*w*), due to insufficient marginal gains via both individual exploitation (*E*; eqn. 3) and the group effects (*G*; eqn. 4). As a consequence, cooperators are selected to disperse more, relative to cheaters. Cheaters may disperse at high levels nonetheless (e.g., case of *n *= 0.1, *k *= 1.2 in Fig. 2c), because in so doing, they lessen the effects of the tragedy of the commons on individual fitness of their kin. In contrast, when the group is dominated by cooperators (high *n*) and public good production is high (*P*), marginal fitness increases with cooperator sedentariness and, due to kin competition (*k*), cheaters are selected to disperse more, relative to cooperators.

### Model 2

**Figure 3:**
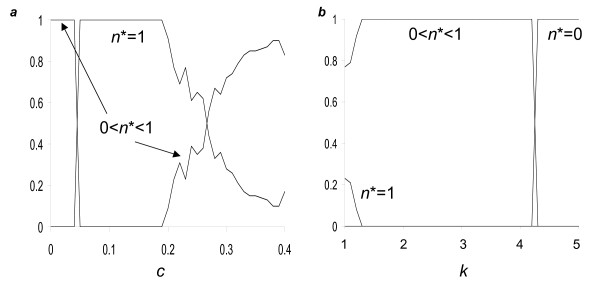
**The fraction of simulations in Model 2 leading to different local optima**. Results based on 100 simulations in which initial levels of *n*, *y*, and *z *are each set to a random number between zero and one, inclusive. These simulations produced one of three equilibria: n* = 0, 0 <n* < 1 or n* = 1. Caption *a *effect of the cost of cooperator dispersal (*c*) with *P *= *Q *= 0.3, *s *= 0.5, *k *= 2, *e *= 0.2; caption *b *effect of effective group size (*k*) with *P *= *Q *= 0.2, *s *= 0.6, *e *= 0.2, *c *= 0.3.

Permitting social evolution introduces the possibility that the frequency of cooperators or cheaters fixes to zero or one, in which case associations (σ) between dispersal and social strategies are irrelevant. We find that depending on parameter combinations, either only a single global optimum is obtained, or two alternative local optima are possible. In the latter case, which state is obtained depends on initial levels of *y, z *and *n *in the numerical simulations. Figure 3 shows the fraction of simulations with random initial levels of *n*, *y *and *z*, achieving either an internal equilibrium (0 <n* < 1), or one with all cooperators (n* = 1), or one with all cheaters (n* = 0) for different costs of cooperator dispersal (*c*; Fig. 3a) and effective group sizes (*k*, Fig. 3b). For simplicity in the analyses below, we employ a single arbitrary starting condition (*n *= *y *= *z *= 0.5).

**Figure 4:**
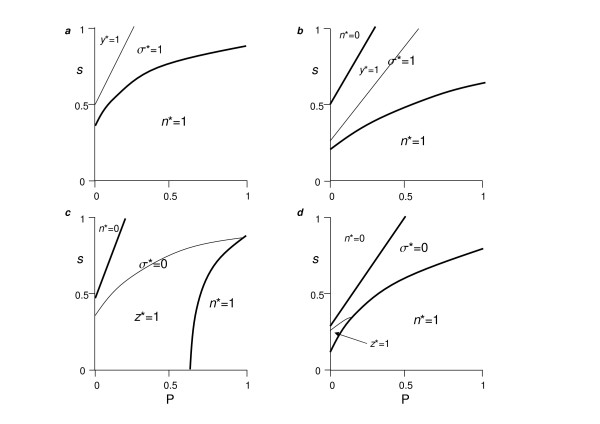
**Locally optimal associations between dispersal and exploitation strategy**. The frequency of dispersal in cooperators (*y*) and cheaters (*z*) evolves, and the frequency of cooperators (*n*) and cheaters (1-*n*) evolves. Initial frequencies in numerical studies: *y *= *z *= *n *= 0.5. As for Figure 1 except caption *a*: *k *= 1.2, *c *= 0.1; caption *b*: *k *= 10, *c *= 0.1; caption *c*: *k *= 1.2, *c *= 0.3; caption *d*: *k *= 10, *c *= 0.3.

We observed four basic outcomes (Fig. 4): (1) fixation of cooperators (n* = 1), (2) fixation of cheaters (n* = 0), or coexistence of cooperators and cheaters with (3) the former only being sedentary (σ * = 0), or (4) the latter only being sedentary (σ * = 1). When σ * = 0 or σ * = 1 (i.e., all cooperators or cheaters sedentary, respectively), we further found outcomes in which all cheaters dispersed (z* = 1) or all cooperators dispersed (y* = 1), respectively. Parameter effects are generally similar to Model 1, but with some notable contrasts.

**Figure 5:**
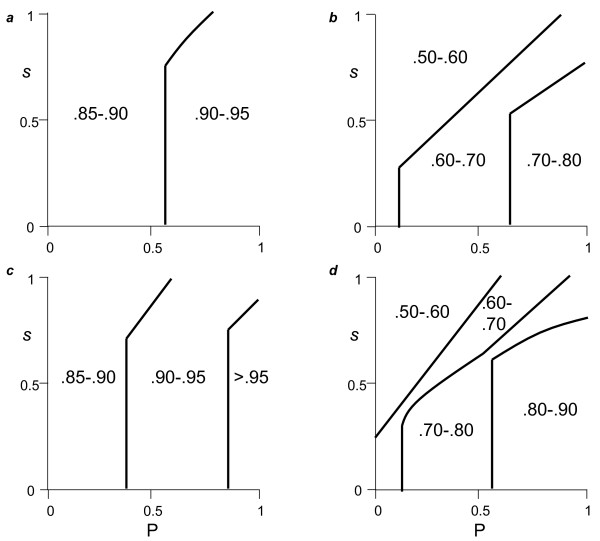
Relatedness, r*, associated with simulations in Figure 4.

Whereas in Model 1, the relative cost of cooperator (*c*) and cheater (*e*) dispersal did not yield a simple threshold condition for optimal outcomes (not shown), it did so for Model 2. We found that when cooperators and cheaters coexisted and *e *> *c*, cooperators dispersed and cheaters did not (i.e., σ * = 1) (Figs. 4a, b). The reverse held when *c *> *e *(Figs. 4c, d). Low effective group size (*k*) increases cooperator persistence (i.e., smaller areas in which n* = 0 in Fig. 4), with the effects on cheater persistence contingent on other parameters (i.e., differences in areas with n* = 0 in Fig. 4). More interestingly, whereas when *e *> *c*, lower *k *shifts the parameter space permitting cooperators and cheaters to coexist and has little effect on the area in which all cooperators disperse (y* = 1), when *c *> *e*, it expands the area of coexistence and that in which all cheaters disperse (z* = 1) (Fig. 4). Finally, relatedness (r*) generally increases with high *P*:*s *ratios, low *k*, and high costs to cooperator dispersal, *c*, with respect to cheater dispersal, *e *(Fig. 5). Interestingly, specialization in dispersal by cheaters and in sedentariness by cooperators tends to associate with high, but not the highest levels of relatedness (cf Figs. 4c, 5c).

**Figure 6:**
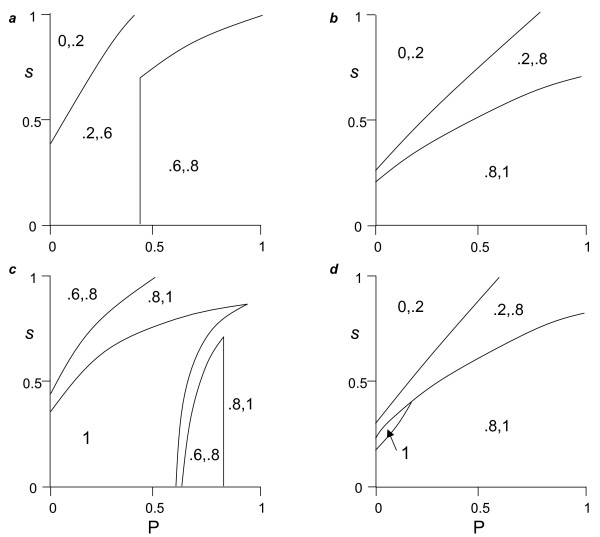
Overall cooperation, Φ = n *(1 - y*)+(1-n*)z*, associated with simulations in Figure 4.

If we define the functional role of a cooperator as contributing to the public good, and that well functioning groups minimize the impact of cheats on the public good, then, trivially, specialization resulting in mobile cooperators and sedentary cheaters corresponds to a non-social, individualistic scenario, and cannot be considered a group related phenomenon. There are however two ways in which the impact of cheaters on the commons can be reduced: either 1-n* decreases and/or z* increases. Figure 6 presents the effects of model processes on overall cooperation, defined as Φ = n* (1-y*) + (1-n*) z*. We see that although high levels of Φ are generally promoted for high *P*:*s *ratios, perfect overall cooperation (Φ = 1) is most readily obtained at low *k *and intermediate *P*:*s *ratios (e.g. Fig. 6c).

## Discussion

Our results are in broad agreement with the tenets of kin selection theory for explaining dispersal [45,81,82] and the maintenance of cooperative behaviors [83-85]. Specifically, we found that dispersal specialization leading to high levels of overall cooperation (Φ) is promoted by sufficient benefit to cost ratios (*P *: *s*) of cooperation and by kin selection (low *k*). The one apparent discrepancy to previous theory is, whereas higher benefit (*P*) to cost (*s*) ratios promote cooperation, higher kin selection (low *k*) was sometimes observed to reduce the relative frequency of cooperators (n*) (cf. Figs. 4c, d). This can be explained if we consider cheaters dispersing from the group as a type of cooperative behavior. Dispersing cheaters are effectively 'cooperative' because of the incurred individual cost of dispersal (*e*), and the benefits to the group in having less negative impact on the commons (*Q*) (cf. Figs 4c, d with Figs. 6c, d). Moreover, we found that partial or total specialization of otherwise somatic cheats as dispersing germ line occurred without the need for costly modifiers [86] or the repression of cheaters [[87,88], but see [22]], suggesting that the mechanism identified here is applicable to a wider range of organisms where these mechanisms do not sufficiently reduce somatic cheating, or cannot evolve. Conversely, control mechanisms such as rewarding and punishment, which might be operating in many systems (see examples in Additional file 1), do not preclude the functionality of the mechanism demonstrated in this study (cf. Model 1).

The examples presented in Table S1 (see Additional file 1) and our theoretical findings suggest a common conceptual and mechanistic foundation for the evolution of cooperation and individual functional specialization within groups (e.g., multicellularity). Most of the empirical examples share the feature that cooperators are less dispersive than more competitive individuals. For instance, low dispersal coincides with physical binding in bacteria that generate biofilms as a public good by polymer production [79,89] (but see ref. [90] for an alternative interpretation), with alloparental care of offspring in cooperative breeding [91], or with complete genetic altruism in certain eusocial insects [92]. It is worth noting that a consistent differentiation of roles regarding sedentariness and dispersal in relation to cooperation and cheating may be much more common in nature than currently believed (e.g. [93]). Because there is no prior formal theory predicting such a relationship, empirical research on this issue is rare and suitable data are therefore scant. We stress that our theory does not elucidate the precise evolutionary pathway leading to complete multicellularity [16,77], but rather assesses the forces promoting or forestalling different levels of specialization of cooperators and cheaters as functional germ line and soma. As such, the observations of biased dispersal in Table S1 (see Additional file 1) have alternative explanations, including forced eviction [94] and individual-based habitat selection [95]. Experimental (e.g., [79,80,96-98]), phylogenetic (e.g., [6]), and theoretical (e.g., [13,22] and see discussion below) approaches are fruitful avenues to explore alternative explanations and pathways.

Transitions in individuality and social complexity are generally thought to require some form of reduction in genetic variance during the reproductive process [20,77]. Genetic heterogeneity can emerge from many sources [99], and the recursive equation 6 in our framework greatly simplifies these, only explicitly including the effects of dispersal. Our results confirm the importance of relatedness in achieving multicellularity, but also show that the highest levels of relatedness did not necessarily yield full specialization of cooperators or cheaters as dispersers, and that complete specialization could occur at relatedness levels as low as 0.7 (Fig. 4d). As such, our findings could extend to some systems in which groups are formed by the initial aggregation of non-kin [10,74,87,99]. Further study is needed to explore this prediction in detail, since our model did not explicitly account for different lineages, and as such we do not know how spatial heterogeneities in relatedness might influence our results [100].

Our findings have precedent, both in the study of symbiotic associations, and investigations of cooperation within species. With regard to host-parasite and symbiotic interactions, previous research has considered how parasite virulence (which is analogous to cheaters exploiting cooperative groups) may evolve spatially (e.g., [101]; for reviews see [102,103]). In the case of horizontal transmission in parasites, which is analogous to the level of dispersal in our model (see also [73]), theory generally predicts that increased horizontal transmission (*z *in our model) associates with higher parasite virulence (Q (1-*n*) in our model) [103]. Despite allowing for relatedness between potential cooperators and cheats we have a comparable finding, whereby an increasing tragedy of the commons pushes cheating individuals to disperse; this is both because of increased individual fitness opportunities through dispersal (*z*) and increased inclusive fitness through lowered group effects for those related individuals that do not disperse (Q(1-*n*) (1-*z*)).

In a model investigating cooperation in spatially viscous environments, van Baalen and Rand [45] suggested that non-altruists should disperse more readily than altruists and hypothesized that this could be viewed as a transition towards multicellularity. Koella [104] studied the independent dispersal of altruists and of cheaters in a spatially explicit setting and found that a polymorphism could arise in which altruists dispersed and interacted locally, whereas cheaters evolved longer dispersal distances and exploited altruistic clusters. Hamilton and Taborsky [95] showed that when the propensities to cooperate by generalized reciprocity and to disperse evolve independently, under a wide range of conditions either cooperation or defection is associated with dispersal, depending on the probability of finding new groups and on the costs of being alone. Over most of the range of mobility costs examined, cooperation was negatively correlated with mobility, while defection was not. Ultimately, this leads to assortment between altruists and defectors in the population (see also [105]), which secondarily can generate group selection effects [106,107]. Hamilton and Taborsky [95] did not check for linkage effects, however. In another study of the joint evolution of altruism and mobility, Le Galliard and coworkers [108] found that more altruism enhancing local aggregation can select for increased mobility. The synergistic selective interaction between altruism and mobility may cause dispersal to be considerably higher than that predicted in a purely selfish population, if altruism costs accelerate slowly and mobility costs are moderate. However, their model did not reveal a polymorphism to occur between selfish-mobile and altruistic-sessile phenotypes as found so often in nature, from microbes and unicellular algae to mammals (e.g. [18,32,109]; Additional file 1). Queller [10] argued that the resolution of within-organism conflicts could occur if an altruism allele is expressed conditional on the environment, the altruistic act being an individual removing itself from the germ line in order to perform an enhanced somatic activity. Rainey [110] verbally proposed an idea similar in some respects to these studies, in which group selection acts to promote the functional separation of germ and soma in bacterial biofilms through the dispersal of cheats (see also [79]). Finally, Michod [14] showed how the specialization of lower level units into germ and soma could be associated with the transfer of fitness from lower units to the new higher individual. A critical feature of his model is the tradeoff between the viability and fecundity of lower level units, which, for convex relationships, creates disruptive selection for cooperative germ and soma. Our study, whilst generating congruent results, is to our knowledge the first to demonstrate that the evolution of lower level units based on their effects on the commons can yield dispersal specialization, one of the precursors for selection at the group level and the evolution of full multicellularity.

## Conclusion

Our results suggest that the establishment of trait linkage between dispersal and the propensity of within-group cheating may be a general phenomenon promoting complex social organization and multicellularity. Importantly, we cautiously suggest this should be operative regardless of whether groups ever achieve higher levels of individuality, because selection on individual components will always tend to increase exploitation, and stronger group structure will tend to increase overall cooperation through kin selected benefits [42,84]. Partial or full reduction in the negative effects of cheaters on the commons through their specialization as dispersers offers partial solutions to two problems: the evolution of cooperation in social groups and the origin of the specialization of germ and soma in multicellular organisms. Our model is, nevertheless, a highly simplified caricature of real systems and future theoretical and empirical study is needed to explore its robustness.

## Authors' contributions

MEH conceived the study, developed and analyzed the model and wrote the manuscript. DJR participated in the design of the study, developed the model and participated in writing the manuscript. MT participated in the design of the study, constructed Table S1, and participated in writing the manuscript. All authors read and approved the final manuscript.

## Supplementary Material

Additional file 1 **(Table S1)**. Examples of group formation for which there is some information on dispersal, relatedness and punishment/policing. Click here for file
