# Supplementary material for: The coevolution of cooperation and dispersal in social groups and its implications for the emergence of multicellularity
Source: BMC Evol Biol. 2008 Aug 19;8:238. doi: 10.1186/1471-2148-8-238 (PMC2533331; doi:10.1186/1471-2148-8-238)
Supplement: Additional file 1 — (Table S1). Examples of group formation for which there is some information on dispersal, relatedness and punishment/policing. [file 1471-2148-8-238-S1.doc]

**Table 1. Examples of group formation for which there is some information on dispersal, relatedness and punishment/policing**.

| **Gross taxonomic level** | **Species** | **Public good** | **Cooperators or cooperation performed** | **Cooperator dispersal** | **Defectors or way of cheating** | **Defector dispersal** | **Relatedness Coop./Def.** | **Punishment, policing** | **References** |
| --- | --- | --- | --- | --- | --- | --- | --- | --- | --- |
| Viruses | Plant *RNA-virus* | diffusable intracellular products | complete RNA-virus | via insects | sequester intracellular products | requires presence of cooperators | defective interfering particle(?) | ? | [111, 112] |
| Bacteria | *Escherichia coli* | protection against competitors | production of diffusive bacteriocins | ? | no colicin production | ? | mutant | colicin production | [113-115] |
| Bacteria | *Pseudomonas sp.* | biofilm | polymer production | shearing | no polymer production | planktonic disperser cells | mutant | Apparent niche exclusion | [79, 80, 89, 116, 117] |
| Myxobacteria | *Myxococcus xanthus* | fruiting body | formation of fruiting body, C-signal production, cell autolysis | S-motility (social gliding) | no contribution to fruiting body | A-motility (individual) | High within group relatedness; mutations | ? | [37, 96, 118-121] |
| Yeast | *Sacharomyces cerevisiae* | Sucrose digestion | production of invertase via *SUC2* gene | Free living | deleted *SUC2* gene, no invertase prod. | Free living | Polymorphic SUC genes | k1 killer toxin production | [122 – 125] |
| Slime moulds | *Dictyostelium mucoroides*  *D. discoideum* | stalk for spore  dispersal  stalk for spore  dispersal | production of signals and stalk, adhesion of cooperators  stalk formation | no  no | specialization in spore production  *chtA/FbxA* -mutant: almost pure spore production | yes  yes | mutant, clone chimeras  mutant clone chimaeras | somatic compatibility system  efficiency reduction by competition, DIF-1 secretion | [126-127]  [97, 128-135] |
| Protozoa  1. Flagellata  a) Phyto-monadina  b) Proto-monadina  2. Euciliata  Peritricha | *Volvox carteri, V. aureus*  *Proterospongia haeckeli*  *Zoothamnium arbuscula* | multicellular body, nutrition, locomotion  multicellular body  multicellular colony | somatic cells  flagellated cells moving the colony  feeding zooids,  nutrition | no  no (?)  no | gonidia: specialize in reproduction  amoeboid cells: asexual reproduction  macrozooids:  no feeding | yes  ?  yes | clonal  clonal  clonal (?) | ?  programmed cell death  ? | [1, 18, 136-147]  [148] |
| Porifera | *Spongilla lacustris, Ephydatia sp.,*  *Reniera sp.,*  *Haliclona sp.* | multicellular body, care for gametes & embryos | up to 14 different cell types, various functions | if dissociated or as gemmules | gamete production | yes | clonal or chimeric | allorecognition restraining exploitation after fusion | [56, 149-155] |
| Coelenterata | *Hydractinia spp.*  *Anthopleura elegantissima* | nutrition, protection  nutrition, protection | gastrozooids, dactylozooids,  tentaculozooids  scout, warrior and free-edge polyps | no  no | gonozooids (♂+♀): no feeding and defence  pure reproductive functions | production of dispersing gametes  production of dispersing gametes | clonal  clonal | partner rejection  ? | [156-160]  [161, 162] |
| Bryozoa | *Dendrobeania murrayana* | nutrition, protection | various zooids | no | gonozooids | production of dispersing gametes | clonal |  | [163-166] |
| Urochordata | *Botryllus schlosseri* | gonads & somatic organs | primordial somatic cells | no | primordial germ cells | yes | distinct cell lineages | gametic cell competition | [24, 167-170] |
| Insecta | *Drosophila melanogaster,*  *D. simulans* | eggs | wild-type sperm (fair meiosis) | yes | segregation distortion | yes | one gene difference | genetic suppression of meiotic drive | [171-174] |
| Mammalia | *Mus musculus* | eggs | wild-type sperm (fair meiosis) | yes | transmission ratio distortion by *t* haplotypes | yes | gene complex diff. on chromos. 17 | mitigating effect of other genes | [175-177] |
| **Analogies in higher Metazoan communities** | | | | | | | | | |
| Isoptera | *Cryptotermes secundus* | nutrition, protection | workers, soldiers | no | reproductives | yes | diploid siblings | ? | [178, 179] |
| Thysanoptera | *Oncothrips habrus, O. tepperi* | gall | micropterous soldiers | no | macropterous reproductives | yes | haplodiploid sisters | ? | [180-184] |
| Aphidae | *Pemphigus spyrothecae, P. obesinymphae* | gall | soldiers | as asex. virginoparae | no defence, accelerated development | as adult sexuparae | partly mixed clones | ? | [185-188] |
| Hymenoptera | *Apis mellifera*  *Meliponini* | nutrition, protection  production of highly related females | workers  mother queen (singly mated) | no  no | reproductives,  egg-laying workers  daughter queens (own reprod. lowering colony relatedness) | queens yes,  workers no  yes | haplodiploid sisters  mother-daughter | by workers  by workers | [189-193]  [194, 195] |
| Pisces | *Neolamprologus pulcher* | protection | breeders and brood care helpers | low | reproductive parasitism by mature helpers | high | very low | expulsion | [25, 27, 28, 87, 196-199] |
| Aves | *Corcorax melano-rhamphos* | group membership,  recruitment of allies | breeders and brood care helpers | conditional | deceptive brood care | conditional | usually high | aggression by group members | [200-204] |
| Mammalia | *Heterocephalus glaber,*  *Cryptomys damarensis* | group membership,  protection | breeders and non-reproductives | no | dispersive morph saves effort and accumulates reserves | yes | high | queen punishment of lazy workers | [32-34, 36, 205-210] |

The public good is the action of cooperators, while defectors do not contribute to the public good. The cooperators or the cooperation performed, and the defectors or their way of cheating, are listed together with information about their respective dispersal, the relatedness between cooperators and defectors, and information about potential coercion in the form of punishment or policing. Note that due to difficulties in obtaining equivalent functional assessments of public goods and dispersal across examples, we considered the former to be a behavior resulting in a potential benefit for one or more group members, and the latter to be movement away from the group. Stricter criteria would be necessary for a more conclusive comparison with model predictions, and thus our objective is to highlight possible similarities and differences, based on first approximations for these complex processes. Question marks denote where respective information is unknown.
